# PHENOstruct: Prediction of human phenotype ontology terms using heterogeneous data sources

**DOI:** 10.12688/f1000research.6670.1

**Published:** 2015-07-16

**Authors:** Indika Kahanda, Christopher Funk, Karin Verspoor, Asa Ben-Hur

**Affiliations:** 1 Department of Computer Science, Colorado State University, Fort Collins, CO, 80523, USA; 2 Computational Bioscience Program, University of Colorado School of Medicine, Aurora, CO, 80045, USA; 3 Department of Computing and Information Systems, University of Melbourne, Parkville, Victoria, 3010, Australia; 4 Health and Biomedical Informatics Centre, University of Melbourne, Parkville, Victoria, 3010, Australia

**Keywords:** human phenotype ontology, structured SVM

## Abstract

The human phenotype ontology (HPO) was recently developed as a standardized vocabulary for describing the phenotype abnormalities associated with human diseases. At present, only a small fraction of human protein coding genes have HPO annotations. But, researchers believe that a large portion of currently unannotated genes are related to disease phenotypes. Therefore, it is important to predict gene-HPO term associations using accurate computational methods. In this work we demonstrate the performance advantage of the structured SVM approach which was shown to be highly effective for Gene Ontology term prediction in comparison to several baseline methods. Furthermore, we highlight a collection of informative data sources suitable for the problem of predicting gene-HPO associations, including large scale literature mining data.

## Introduction

In the medical context a phenotype is defined as a deviation from normal morphology, physiology, or behavior ^1^. The human phenotype ontology (HPO) is a standardized vocabulary that describes the phenotype abnormalities encountered in human diseases ^2^. It was initially populated using databases of human genes and genetic disorders such as OMIM ^3^, Orphanet ^4^ and DECIPHER ^5^, and was later expanded using literature curation. The hierarchical structure of the HPO is very similar to that of the Gene Ontology (GO) ^6^, and it too has the structure of a directed acyclic graph (DAG); like GO, more general terms are found at the top, and term specificity increases from the root to the leaves. This implies the “true-path rule”: whenever a gene is annotated with a given term, that implies all its ancestor terms.

**Figure 1.**
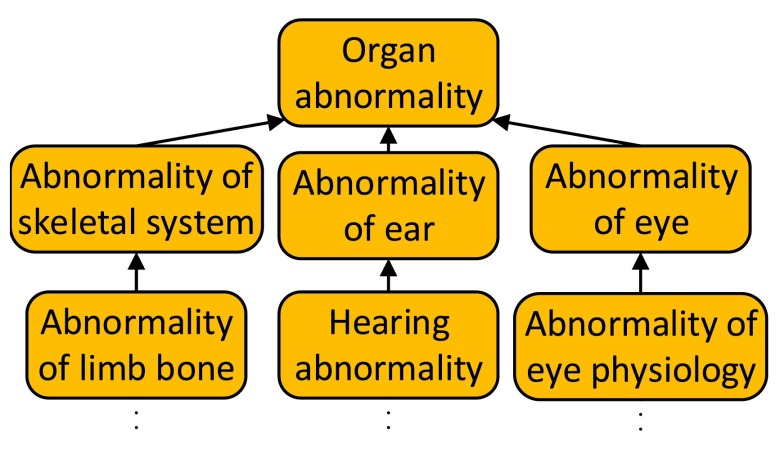
A portion of the Organ abnormality subontology. All HPO parent-child relationships represent “is-a” relationships.

HPO is composed of three subontologies: organ abnormality, mode of inheritance, and onset and clinical course. Organ abnormality is the main subontology which describes clinical abnormalities ( Figure 1). The mode of inheritance subontology describes the inheritance patterns of the phenotypes. The onset and clinical course subontology describes the typical time of onset of clinical symptoms and their speed of progression. The organ abnormality, mode of inheritance and onset and clinical course subontologies are composed of ~10000, 25 and 30 terms respectively. Throughout this paper, the organ abnormality, the mode of inheritance, and the onset and clinical course subontologies will be referred to as the Organ subontology, Inheritance subontology and Onset subontology, respectively.

**Figure 2.**
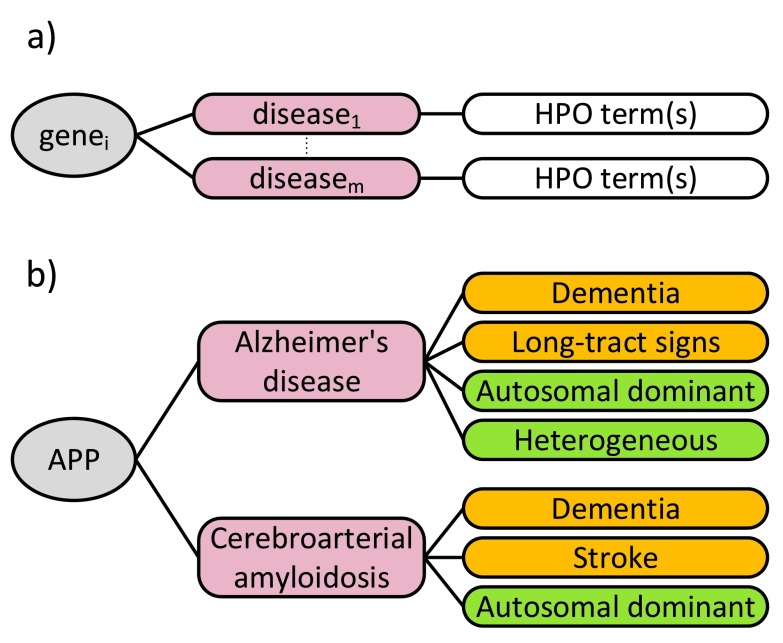
HPO annotations. **a**) general format of annotations: genes are annotated with a set of phenotype terms based on their known relationships with diseases **b**) an example annotation: the amyloid precursor protein (APP) gene is associated with Alzheimer’s disease and cerebroarterial amyloidosis. Therefore, the APP gene is annotated with the set of HPO terms (Organ in orange, Inheritance in green) associated with these diseases.

The HPO web site ( http://www.human-phenotype-ontology.org) provides gene-disease-HPO annotations that can be used for research involving human diseases. Over 50,000 annotations of hereditary diseases are available at the moment. Specifically, the genes are annotated with a set of phenotype terms based on their known relationships with diseases ( Figure 2).

Currently, only a small fraction (~3000) of human protein coding genes are known to be associated with hereditary diseases, and only those genes have HPO annotations at the moment. But researchers believe that there are many other disease-causing genes in the human genome and estimate that another 5000 genes can be associated with phenotypes (Peter Robinson, personal communication, 2014). However, experimentally finding disease-causing genes is a highly resource consuming and difficult task ^7^. Therefore, it is important to explore the feasibility of developing computational methods for predicting gene-HPO associations. While there is a plethora of computational approaches for the related task of prediction of gene-disease associations ^8^, no computational method that directly predicts gene-HPO term associations exists at this time.

## Approach

We define the HPO prediction problem as directly predicting the complete set of HPO terms for a given gene. This problem is a hierarchical multilabel classification (HMC) problem ^9^, as a given gene can be annotated with multiple labels, and the set of labels have a hierarchy associated with them.

The traditional approach for solving HMC problems is to decompose the problem into multiple single label problems and apply independent binary classifiers for each label separately ^10^; however, this approach has several disadvantages. First, independent classifiers are not able to learn from the inter-relationships between the labels. Second, the leaf terms typically have a low number of annotated examples making it difficult to learn an effective classifier. Furthermore, the predicted labels are typically hierarchically inconsistent, i.e. a child term (e.g. Hearing abnormality) is predicted while its parent term (e.g. Abnormality of ear) is not—making it difficult to interpret the predictions. To remedy this problem, an additional reconciliation step of combining independent predictions to obtain a set of predictions that are consistent with the topology of the ontology is required (see e.g. 11 for a discussion of several reconciliation methods that are effective for GO term prediction).

An alternative approach is to use a single classifier that learns a direct mapping from inputs to the space of hierarchically consistent labels; this can be achieved using structured prediction, which is a framework for learning a mapping from inputs to label spaces that have a structure associated with them ^12^. This framework can capture information from the inter-relationships between labels and allows the prediction of a set of labels that are hierarchically consistent, eliminating the need for multiple classifiers, and the need for establishing hierarchical consistency between the predictions. Previously we have shown the effectiveness of modeling the GO term prediction problem using a structured prediction framework in a method called GOstruct ^13, 14^. In this work we demonstrate the effectiveness of this strategy for HPO term prediction using the same methodology, and explore a variety of data sources that are useful for this task, including large scale data extracted from the biomedical literature.

## Methods

### Data

Our models are provided with feature vectors and HPO annotations. Each gene/protein was characterized by several sets of features generated using four data sources: Network, GO, literature and variants, which are described below. We used the UniProt ID mapping service ( http://www.uniprot.org/mapping/) for mapping genes to proteins.

### HPO annotations

Gene-HPO annotations were downloaded from the HPO website ( http://www.human-phenotype-ontology.org). We ignored the global root term (“ALL”) and root terms of the three subontologies. We also removed terms that were not annotated to 10 or more genes. Then we mapped the genes to proteins and generated corresponding protein-HPO annotations (see Table 1).

**Table 1.** Number of genes, unique terms and annotations. The “unique terms” column provides both the number of terms and the number of leaf terms; the “annotations” column provides the number of annotations, as well as their number when expanded using the true-path rule.

Subont.	Genes	Terms	Annotations
Organ	2,768	1,796/1,337	213k/60k
Inheritance	2,668	12/10	3.6k/3.3k
Onset	926	23/20	1.7k/1.4k

### Network

We extracted protein-protein interactions and other functional association network data (i.e. co-expression, co-occurrence, etc.) from BioGRID 3.2.106 ^15^, STRING 9.1 ^16^ and GeneMANIA 3.1.2 ( http://pages.genemania.org/data/) databases.

The BioGRID database provides protein-protein interaction networks acquired from physical and genetic interaction experiments. STRING provides networks based on several different evidence channels (co-expression, co-occurrence, fusion, neighborhood, genetic interactions, physical interactions, etc.). We combined edges from the two databases by taking the union of interactions from BioGRID and STRING and represented each gene by a vector of variables, where component *i* indicates if the corresponding protein interacts with protein *i* in the combined network.

The GeneMANIA website ( http://pages.genemania.org/data/) provides a large number of protein-protein interaction/association networks generated using several types of evidence: co-expression, co-localization, genetic interactions, physical interactions and predicted interactions. A gene is represented by a vector of variables for each network, where component *i* indicates if the corresponding protein interacts with protein *i* with respect to that particular network.

### Gene Ontology

We extracted GO ^6^ annotations from the GO web site ( http://www.geneontology.org/) and Uniprot-goa ( http://www.ebi.ac.uk/GOA). We excluded all annotations that were obtained by computational methods. A gene is represented as a vector of indicator variables in which variable *i* is 1 if it is annotated with GO term *i*.

### Literature

We used two different sources for generating literature features: abstracts extracted from Medline on 10-23-13 and full-text articles extracted from PubMed Open Access Collection (PMCOA) on 11-06-13. A natural language processing pipeline was utilized to characterize genes/proteins by same-sentence word occurrences extracted from these sources, forming a bag-of-words (BoW) representation for each gene ^17^. First, all words were lower-cased and stop words were removed. Then they were further filtered to keep only the low frequency words (i.e. words that are present only in less than 1% of the proteins in the data). A gene is represented by a vector in which the element *i* gives the number of times the word *i* occurred in the same sentence with that gene/protein.

### Variants

We extracted all the disease variants in the human genome and their associated diseases from UniProt ( http://www.uniprot.org/docs/humsavar). This data provides variants that have been found in patients and the disease-association is reported in literature. We also extracted gene-disease associations from the HPO website. This data associates a protein with diseases that are known to occur when the associated gene is mutated. To generate features from this data, we first extracted for each protein *p _i_* its set of associated diseases ( *D _i_*) from the protein-disease associations. Then we retrieved the set of disease variants ( *V _i_*) associated with all diseases in *D _i_* from the UniProt disease variants data. Finally, each gene was represented by a vector in which element *j* indicates if the variant *j* is in *V _i_*.

### Models

In this work we compare a structured support vector machine approach against several baseline methods: a) binary support vector machines (SVMs) and b) a state-of-the-art HMC method based on decision tree ensembles (Clus-HMC-Ens). In this section we describe PHENOstruct and the two baseline methods. In addition, we assessed the performance of: c) an indirect method that first predicts disease terms for a gene using a structured model and then maps them to HPO terms and d) using OMIM disease terms predicted by PhenoPPIOrth ^18^ followed by mapping the OMIM terms to HPO terms. We describe these two additional methods in the Supplementary material (see section “Additional methods”). All methods except PhenoPPIOrth were provided the same data.

### PHENOstruct

**Figure 3.**
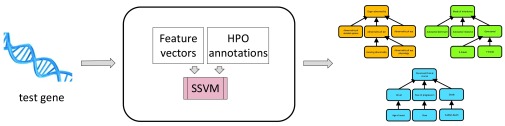
Overview of PHENOstruct. PHENOstruct takes the set of feature vectors and HPO annotations associated with each gene as input for training. Once trained, it can predict a set of hierarchically consistent HPO terms for a given test gene. PHENOstruct is trained on and makes predictions for a single subontology at a time (DAGs belonging to Organ, Inheritance and Onset subontologies are shown in orange, green and blue, respectively).

In earlier work we developed the GOstruct method which uses structured SVMs (SSVM) for GO term prediction ^13^. In this work we apply the same methodology to HPO term prediction and refer to it as PHENOstruct to emphasize the different problem domain. Unlike collections of binary classifiers applied independently at each node of the hierarchy, PHENOstruct predicts a set of hierarchically consistent HPO terms for a given gene ( Figure 3). More specifically, PHENOstruct learns a compatibility function that models the association between a given input and a structured output ^12^, in this case the collection of all hierarchically consistent sets of HPO terms. Let *𝒳* be the input space where genes are represented and let *𝒴* be the space of labels. The set of HPO terms associated with a given gene is collectively referred to as its (structured) label. *𝒴* represents each HPO subontology in a vector space where component *i* represents term *i*. Given a training set ${\left\{(x_{i},\;y_{i})\right\}}_{i=1}^{n}$ where *x _i_*∈ *𝒳* and *y _i_*∈ *𝒴*, the *compatibility* function *f* : *𝒳* × *𝒴* → *ℛ* maps input-output pairs to a score that indicates how likely is a gene *x* to be associated with a collection of terms represented by *y*. The predicted label *ŷ* for an unseen input *x* can then be obtained by using the argmax operator as *ŷ* = argmax _*y*∈ *𝒴 _c_*_ *f*( *x*, *y*) where *𝒴 _c_* ⊂ *𝒴* is the set of all candidate labels. In this work we use the combinations of all terms in the training set as the set of candidate labels *𝒴 _c_*.

**Figure 4.**
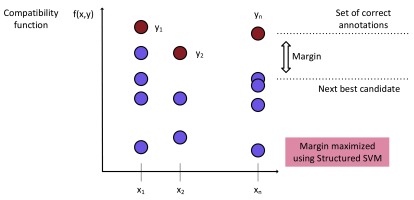
Visual interpretation of the structured prediction framework. The compatibility function, which is the key component of the structured prediction framework, measures compatibility between a given input and a structured output. The compatibility function of the true label (correct set of HPO annotations) is required to be higher than that of any other label. and the difference between these two scores (margin) is maximized.

In order to obtain correct classification, the compatibility value of the true label (correct set of HPO annotations) of an input needs to be higher than that of any other candidate label ( Figure 4). PHENOstruct uses structured SVM (SSVM) training where this is used as a (soft) constraint; it tries to maximize the margin, or the difference between the compatibility value for the actual label and the compatibility for the next best candidate ^12^. In the structured-output setting, kernels correspond to dot products in the joint input-output feature space, and they are functions of both inputs and outputs. PHENOstruct uses a joint kernel that is the product of the input-space and the output-space kernels:

$$K((x_{1},\;y_{1}),\;(x_{2},\;y_{2}))\;=\;K_{𝒳}(x_{1},\;x_{2})K_{𝒴}(y_{1},\;y_{2})\text{.}$$

The motivation for this form is that two input/output pairs are considered similar if they are similar in both their input space features and their labels; the output space kernel, for which we use a linear kernel between label vectors, captures similarity of the annotations associated with two genes; the input space kernel combines several sources of data by the addition of multiple input-space kernels, one for each data source. Each kernel is normalized according to

$$K_{norm}(z_{1},\;z_{2})\;=\;K(z_{1},\;z_{2})/\sqrt{K(z_{1},\;z_{1})K(z_{2},\;z_{2})}$$

before being used with the joint input-output kernel. The Strut library ( http://sourceforge.net/projects/strut/) with default parameter settings was used for the implementation of PHENOstruct.

### Binary SVMs

As a baseline method we trained a collection of binary SVMs, each trained on a single HPO term. Binary SVMs were trained using the PyML ( http://pyml.sourceforge.net) machine learning library with default parameter settings. We used linear kernels for each set of input space features.

### Clus-HMC-Ens

Clus-HMC-Ens is a state-of-the-art HMC method based on decision tree ensembles which has been shown to be very effective for GO term prediction ^19^. In our study, we provide exactly the same set of features used with PHENOstruct as input to Clus-HMC-Ens and use parameter settings that provided the best performance for GO term prediction ( https://dtai.cs.kuleuven.be/clus/hmc-ens/). The number of bags used was 50 for the Inheritance and Onset subontologies; 10 bags were used for the Organ subontology because of the large running times for this subontology.

### Evaluation

Classifier performance was estimated using five-fold cross-validation. Since typically scientists/biologists are interested in knowing the set of genes/proteins associated with a certain HPO term, we primarily use a *term-centric* measure for presenting results. Term-centric measures average performance across terms as opposed to *protein-centric* measures which average performance across proteins as described elsewhere ^20^. More specifically, we use the macro AUC (area under the receiver operating curve), which is computed by averaging the AUCs across HPO terms. For comparing performance across classifiers, p-values were computed using paired t-tests. Additionally, we report performance in terms of several protein-centric measures (precision, recall, F-max) in the Supplementary material ( Table S3 and Table S4). Definitions of all performance measures are given in the Supplementary material. PHENOstruct assigns a confidence score to each predicted HPO term, which is computed using the compatibility function as described elsewhere ^14^. The onset and clinical course subontology includes terms such as *pace of progression, age of onset* and *onset* which are only used for grouping terms. We ignore these grouping terms when computing performance.

## Results and discussion

### PHENOstruct performance

As illustrated in Table 2, PHENOstruct significantly outperforms Clus-HMC-Ens and the binary SVMs in the Organ and Onset subontologies. This suggests that modeling the HPO prediction problem as a structured prediction problem is highly effective. It is interesting to note that the biggest improvement of PHENOstruct over binary SVMs is seen in the Organ subontology. Given its very large number of terms, as well as the deep hierarchy, this further confirms the value of the structured approach. PHENOstruct outperforms binary SVMs in the Inheritance and Onset subontologies but to a lesser extent than in the Organ subontology because they are far less complex than the Organ subontology. We note that the two methods that first predict OMIM terms, which are then mapped to HPO terms performed poorly (see details in the Supplementary material). It is also interesting to see that Clus-HMC-Ens performs worse than binary SVMs with respect to macro AUC ( Table 2) but performs slightly better than binary SVMs according to protein-centric F-max ( Table S3).

**Table 2.** PHENOstruct vs. other methods. Performance across the three HPO subontologies for PHENOstruct, binary SVMs and Clus-HMC-Ens measured using the macro AUC. P-values provide the significance level for the difference between the corresponding method and PHENOstruct.

Subont.	Terms	Method	AUC	P-value
Organ	1,796	Binary SVMs	0.66	1.7E-262
		Clus-HMC-Ens	0.65	0.0E+00
		PHENOstruct	**0.73**	—
nherit.	12	Binary SVMs	0.72	2.2E-01
		Clus-HMC-Ens	0.73	7.3E-01
		PHENOstruct	**0.74**	—
Onset	23	Binary SVMs	0.62	4.4E-03
		Clus-HMC-Ens	0.58	3.3E-05
		PHENOstruct	**0.64**	—

**Table 3.** Performance of PHENOstruct in the Inheritance subontology. The average macro AUC for the Inheritance subontology is 0.74. Terms are displayed in ascending order of frequency.

Name	Freq.	Depth	AUC
Multifactorial inheritance	15	1	0.54
Polygenic inheritance	15	2	0.54
Mitochondrial inheritance	41	1	0.98
Sporadic	52	1	0.61
Somatic mutation	61	1	0.76
X-linked dominant inheritance	62	3	0.83
X-linked recessive inheritance	111	3	0.77
Heterogeneous	148	1	0.69
Gonosomal inheritance	198	1	0.80
X-linked inheritance	198	2	0.80
Autosomal dominant inherit.	1096	1	0.78
Autosomal recessive inheri.	1665	1	0.73

PHENOstruct’s average AUC for the Organ and Inheritance subontologies are 0.73 and 0.74, respectively. Even though the Organ subontology is a far more complex subontology than the Inheritance subontology (with thousands of terms and 13 levels as opposed to tens of terms and only 3 levels) they show similar performance. The Onset subontology is the hardest to predict accurately, with an average AUC of 0.64. Only six Onset subontology terms have individual AUCs above 0.7 ( Table 4).

**Table 4.** Performance of PHENOstruct in the Onset subontology. The average macro AUC for the Onset subontology is 0.64. Terms are displayed in ascending order of frequency.

Name	Freq.	Depth	AUC
Late onset	11	4	0.70
Neonatal death	14	2	0.54
Sudden death	14	2	0.50
Nonprogressive disorder	15	2	0.82
Stillbirth	21	2	0.67
Death in childhood	23	2	0.65
Neonatal onset	23	3	0.64
Rapidly progressive	33	2	0.50
Childhood onset	41	3	0.62
Death in infancy	44	2	0.70
Incomplete penetrance	58	2	0.61
Juvenile onset	90	3	0.70
Slow progression	95	2	0.62
Adult onset	98	3	0.71
Death	111	1	0.61
Variable expressivity	132	2	0.66
Congenital onset	135	3	0.60
Progressive disorder	141	2	0.70
Infantile onset	245	3	0.66
Phenotypic variability	310	1	0.65

Even though PHENOstruct outperforms the baseline methods, there is much room for improvement, especially in the Onset subontology. The small number of annotated genes in this subontology ( Table 1) makes it difficult to train an effective model while the incomplete nature of the current gold standard used for evaluation tends to underestimate performance of classifiers ^21^. See section for a detailed analysis of false positives.

**Figure 5.**
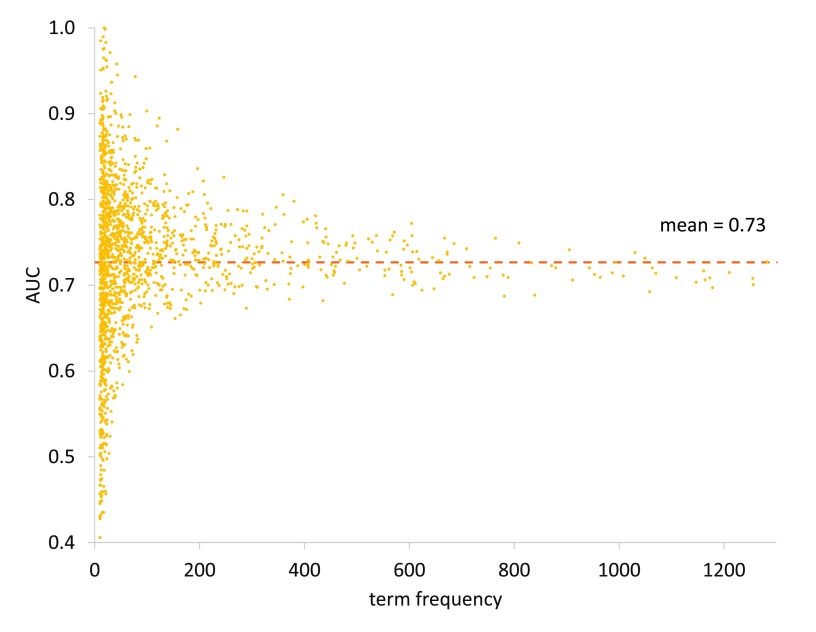
Performance of PHENOstruct in the Organ subontology. Performance for each term is displayed using AUC against its frequency. The average AUC for the Organ subontology is 0.73.

**Figure 6.**
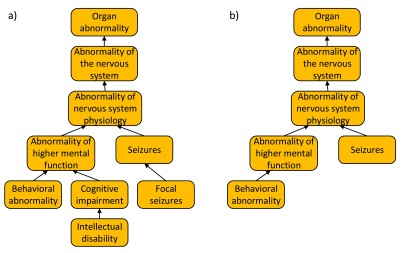
Example of experimental and predicted annotations. **a**) experimental annotation of protein P43681 **b**) PHENOstruct’s prediction for P43681 (protein-centric precision and recall for this individual protein is 1.0 and 0.62, respectively).

In general, Organ subontology terms with few annotations show a mix of both high and low performance as illustrated in Figure 5. This suggests that PHENOstruct is not necessarily affected by the frequency of the terms. But, terms with more annotations tend to show moderate performance. See Figure 6 for an example of experimental and predicted annotations (Organ subontology) for a protein. It is interesting to note that “polygenic inheritance” and its parent term “mulifactorial inheritance” have the lowest number of annotations as well as the lowest individual AUCs in the Inheritance subontology (see Table 3). These are the two terms with the lowest AUC with binary SVMs as well (see Table S6). It is not surprising that these two terms have lower accuracy because each describes inheritance patterns that depend on a mixture of determinants. Moreover, the diseases inherited in this manner – termed complex diseases – are not as well characterized and annotated compared to Mendelian/single gene diseases. On the other hand, the mitochondrial inheritance term has an exceptional AUC of 0.98. It is also the term with the highest AUC with the binary SVMs as well (see Table S6). The human mitochondrial DNA was the first significant part of the human genome to be fully sequenced, two decades before the completion of the human genome project ^22^. Due to this, and the relative ease of sequencing the mitochondrial genome ^23^, diseases caused by mutations in human mitochondrial DNA have been reported very early ^24, 25^. It is likely that this well-studied nature of mitochondrial DNA leads to the high performance of the mitochondrial inheritance term.

As a potential improvement to PHENOstruct we explored an approximate inference algorithm that replaces computation of the most compatible label by looping overall combinations of labels that occur in the training data with a dynamic programming algorithm that performs approximate evaluation of all possible combinations of hierarchically consistent labels. However, this led to a slight decrease in performance, showing the advantage of considering only the biologically relevant combinations. Further research should consider other alternatives.

All experiments were performed on Linux running machines with 8 cores (64-bit, 3.3GHz) and 8GB memory. Combined running times for performing five-fold cross-validation for all three subontologies are: binary SVMs: 55 hours, Clus-HMC-Ens: 825 hours and PHENOstruct: 90 hours.

### Effectiveness of individual data sources

We performed the following set of experiments in order to identify the most effective data sources for HPO prediction using PHENOstruct. First, to identify the individual effectiveness of each source, we performed a series of experiments in which we provided features generated from a single source of data at a time as input to PHENOstruct. Then to understand how much each data source is contributing to the overall performance we conducted leave-one-source-out experiments.

**Figure 7.**
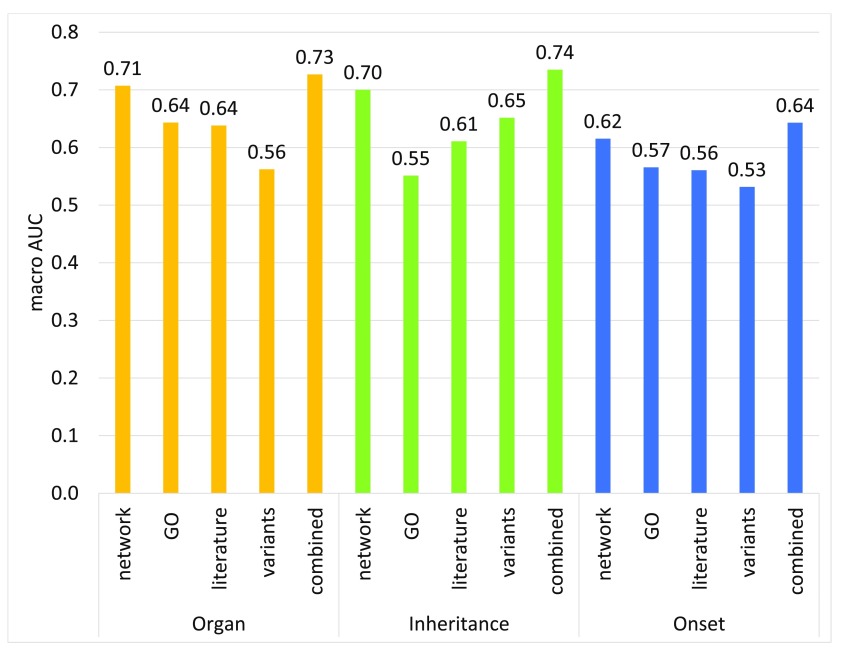
Performance of PHENOstruct with individual data sources. Results are shown for each source of data: network (functional association data); Gene Ontology annotations; literature mining data; genetic variants; and the model that combines all features together.

**Figure 8.**
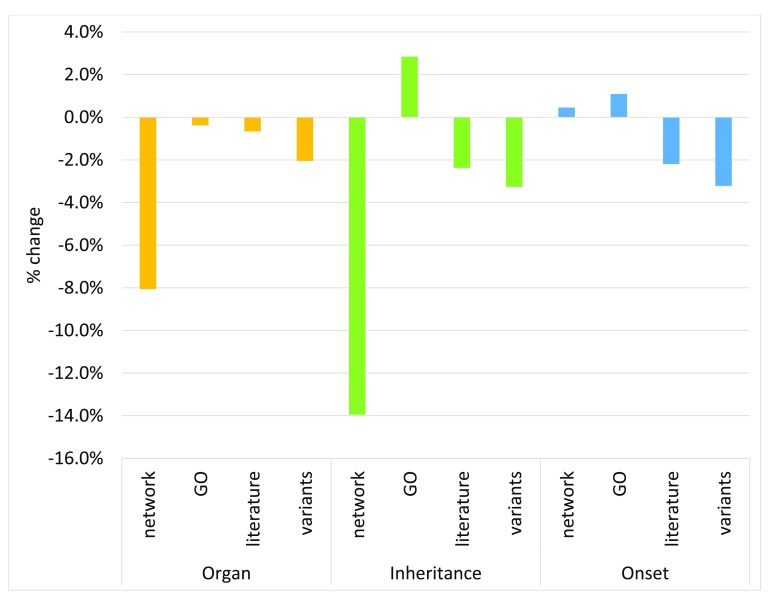
Performance of PHENOstruct in leave-one-source-out experiments (measured by the % change in macro AUC by leaving out a single selected source relative to its macro AUC obtained using all data sources; negative % change means the performance dropped after leaving out the particular source of data).

In all three subontologies, network data is the most informative individual data source as illustrated in Figure 7. Moreover, it is by far the main contributor to the overall performance both in the Organ and Inheritance subontologies ( Figure 8). This is intuitive because if two genes/proteins are known to be interacting and/or active in the same pathways it leads to association with the same/similar diseases/phenotypes.

Although the genetic variant features provide the lowest performance in the Organ and Onset subontologies, leaving out variant data hurts the overall performance noticeably in all three subontologies as can be seen in Figure 8. This suggests that variant data are very useful especially as a complementary dataset to the others. Moreover, we found that variant data are very effective for predicting cancer-related terms in the Organ subontology (see Table S1).

It is very encouraging to see that the literature data with a simple BoW representation by itself is very informative ( Figure 7) and leaving out literature features shows considerable performance drop in the other two subontologies ( Figure 8). In an analysis of the SSVM weight vector, we found that the majority of the most important tokens extracted from literature consist of names of proteins, genes and diseases (see Table S2).

We also considered an alternative representation of the literature data where a gene is represented by a vector in which the element *i* gives the number of times the word *i* occurred in the same sentence with that particular gene/protein divided by the total number of unique genes/proteins that word co-occurred with. This representation is analogous to the TFIDF (term frequency ∗ inverse document frequency) representation typically used in information retrieval and text mining ^26^. However, these features led to slight deterioration of performance in all three subontologies (macro AUCs 0.60, 0.58 and 0.56 for Organ, Inheritance and Onset subontologies, respectively).

Although GO features provide the second best individual performance both in the Organ and Onset subontologies ( Figure 7), their contribution to the overall performance is very minimal ( Figure 8). In fact leaving out GO features increases the overall performance in the Inheritance and Onset subontologies. The incompleteness of GO annotations may have contributed towards this.

Finally, the combination of all the features provides higher performance than individual feature sets in all three subontologies as can be seen in Figure 7. However, leaving out GO features in the Inheritance and Onset subontologies, led to improved performance, suggesting that not all sources contribute to the overall performance. This shows that the selection of data sources must be performed carefully in order to find the optimal combination of sources for each subontology.

### Validating false positives

Like other biological ontologies, the HPO is incomplete due to various factors such as slowness of the curation process ^27^. In other words, the set of HPO annotations we considered as the gold standard does not fully represent all the phenotypes that should be associated with the currently annotated genes; this leads to performance estimates that underestimate the true performance of a classifier ^21^. To explore this issue, we selected 25 predictions made by PHENOstruct which were considered false positives according to the current gold standard and looked for evidence in the current biomedical literature that can be used as evidence for those predictions. For 14 of those predictions we were able to find supporting evidence. The details of the complete validation process are given in the Supplementary material.

## Conclusions and future work

This is the first study of directly predicting gene-HPO term associations. We modeled this problem as a hierarchical multi-label problem and used the SSVM framework for developing PHENOstruct. Our results demonstrate that using the SSVM is more effective than the traditional approach of decomposing the problem into a collection of binary classification problems. In our experiments we evaluated several types of data which were found to be informative for HPO term prediction: networks of functional association, large scale data mined from the biomedical literature and genetic variant data.

There are several ways in which this work can be extended. For the literature data we used a simple BoW representation. An alternative is to try and extract gene-HPO term co-mentions directly; in the context of GO term prediction we have found that both approaches lead to similar overall performance ^17^. However, co-mentions have the added value that they are easy to verify by a human curator. Another source of information that can be utilized is semantic similarity of HPO terms to other phenotypic ontologies such the mammalian phenotype ontology, which is currently used for annotating the rat genome ^28^. Finally, exploring the effectiveness of combining all three subontologies, as opposed to treating them as three independent subontologies as we have done here, is also worth exploring.

Although PHENOstruct outperformed the baseline methods, there is considerable room for improvement in all three subontologies. While some improvement can likely be obtained as described above, its performance will also improve as the number of HPO annotations increases. HPO is a relatively new ontology that will likely see substantial growth in the coming years, which will help in improving the accuracy of computational methods that contribute to its expansion.

## Data and software availability

Zenodo: Data and software associated with PHENOstruct:Prediction of human phenotype ontology terms using heterogeneous data sources, 10.5281/zenodo.18764 ^29^

## Figures and Tables

**Table S1.** The Organ subontology terms that are well-predicted by variant features with PHENOstruct.

HPO ID	HPO term	Freq	Depth	AUC	Cancer-related
HP:0006846	Acute encephalopathy	15	5	1.00	No
HP:0006965	Acute necrotizing encephalopathy	15	6	1.00	No
HP:0003287	Abnormality of mitochondrial metabolism	16	4	1.00	No
HP:0008316	Abnormal mitochondria in muscle tissue	16	5	1.00	No
HP:0012103	Abnormality of the mitochondrion	16	3	1.00	No
HP:0002141	Gait imbalance	14	5	1.00	No
HP:0000148	Vaginal atresia	19	7	1.00	No
HP:0001827	Genital tract atresia	19	4	1.00	No
HP:0002862	Bladder carcinoma	22	5	1.00	Yes
HP:0006740	Transitional cell carcinoma of the bladder	22	6	1.00	Yes
HP:0009725	Bladder neoplasm	22	4	1.00	Yes
HP:0010784	Uterine neoplasm	29	7	0.98	Yes
HP:0002672	Gastrointestinal carcinoma	23	6	0.98	Yes
HP:0006716	Hereditary nonpolyposis colorectal carcinoma	23	7	0.98	Yes
HP:0006749	Malignant gastrointestinal tract tumors	23	5	0.98	Yes
HP:0010747	Medial flaring of the eyebrow	14	6	0.98	No
HP:0002891	Uterine leiomyosarcoma	20	8	0.98	Yes
HP:0100243	Leiomyosarcoma	20	4	0.98	Yes
HP:0004481	Progressive macrocephaly	18	6	0.97	No
HP:0007707	Congenital primary aphakia	14	7	0.97	No
HP:0100834	Neoplasm of the large intestine	33	6	0.97	Yes
HP:0006519	Alveolar cell carcinoma	13	6	0.97	Yes
HP:0100552	Neoplasm of the tracheobronchial system	13	5	0.97	Yes
HP:0009806	Nephrogenic diabetes insipidus	15	3	0.95	No
HP:0100273	Neoplasm of the colon	15	7	0.95	Yes
HP:0005584	Renal cell carcinoma	28	6	0.94	Yes
HP:0003002	Breast carcinoma	20	3	0.94	Yes
HP:0006753	Neoplasm of the stomach	39	5	0.94	Yes
HP:0100013	Neoplasm of the breast	31	2	0.92	Yes
HP:0004808	Acute myeloid leukemia	22	5	0.92	No
HP:0003006	Neuroblastoma	19	7	0.91	Yes
HP:0004376	Neuroblastic tumors	19	6	0.91	Yes
HP:0002370	Poor coordination	18	6	0.90	No
HP:0010786	Urinary tract neoplasm	49	3	0.90	Yes
HP:0000142	Abnormality of the vagina	27	6	0.90	No
HP:0001413	Micronodular cirrhosis	14	5	0.90	No
HP:0009726	Renal neoplasm	47	5	0.90	Yes

Terms are listed in the ascending order of their individual AUCs. 21 out of the 37 (57%) terms well-predicted Organ subontoloy terms by the variant data are terms related to cancer.

**Table S2.** The top-100 literature features with respect to the 8 HPO terms that have individual AUCs equal to or above 0.9 in the organ subontology.

Category	Tokens
proteins/protein complexes	cx32, kisspeptin, -308, t308, smn2, ns5, trap-positive, mpp+-induced, 1-methyl-4-phenylpyridinium, tnf-alpha-mediated, tnf-alpha-stimulated, tnf–mediated, ink4a/arf, ns4b, hmsh6, fukutin, cdtb, ns5b, apoai, tnf–stimulated, ns4a, tnf-alpha-, rhbmp-2, tnf-alpha-treated, frataxin, ki-ras, connexin32, tcdb, recql4, =-galcer, tyrosinase-related, hpms2, her4, cd40-cd40l, lmp2a, ryrs, mg2+-atpase, ews-fli1, abeta42, fancc, p40phox, her1, bdnf-induced, trap+, gfap-ir, daf-16/foxo, hdl3, -238, [tnf-alpha], cd40/cd40l, tnf–treated, anti-ngf, tep1, recq, nt-4, pfemp1, zo-2, nphp1, tnf-alpha-dependent, pomt1, igm-positive, apoa-ii, p110alpha, fancf, tbx4, anti-cd40l, igg
genes	hmsh2, cx26, fkrp, smn1, cln3, nphp4, mn1, nnt, apex2, akt-2
pathways	ras/raf/mek/erk, pi3k-akt-mtor
diseases/phenotypes	cmt1a, hnpp, hdl2, cln2, hpp, fmf, rtt, hnpcc, charcot-marie-tooth, amenorrhea, rett, anticardiolipin
misc.	sheldrick, shelxl97, bruker, farrugia, ortep-3, platon, shelxs97, spek, sgdid, wlds, caii, aoa, tdf, crysalis, wingx, amf

The union set of the top-100 literature features with respect to the 8 HPO terms that have individual AUCs equal to or above 0.9. It is composed of 107 unique tokens. The token “-308” and “t308” in the “proteins/protein complexes” category are due to mis-tokenization of “miR-308”. Similarly, “-238” in the same category is due to mis-tokenization of “BQ-23”. Also “=-galcer” in the same category originated from *α*-galcer and *β*-galcer due to mis-handling of UTF characters *α* and *β*.

**Figure S1.**
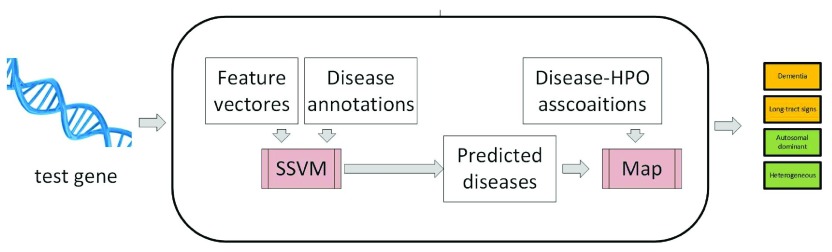
SSVM → disease → HPO method. This method takes feature vectors and disease annotations associated with each gene as the input for training a SSVM model. Then, it predicts diseases for unseen genes using the learned model. Subsequently, the predicted scores for disease terms are directly transferred to all the HPO terms associated with those diseases.

**Table S3.** Comparison between PHENOstruct and other methods.

Subontology	Method	F-max	Precision	Recall	mac-AUC
Organ	PhenoPPIOrth	0.20	0.27	0.15	0.52
	Struct->Dis->HPO	0.23	0.16	0.41	0.49
	Binary SVMs	0.35	0.32	0.40	0.66
	Clus-HMC-Ens	0.41	**0.39**	0.43	0.65
	PHENOstruct	**0.42**	0.35	**0.56**	**0.73**
Inheritance	PhenoPPIOrth	0.12	0.16	0.10	0.55
	Struct->Dis->HPO	0.11	0.07	0.25	0.46
	Binary SVMs	0.69	0.62	0.78	0.72
	Clus-HMC-Ens	0.73	0.64	**0.84**	0.73
	PHENOstruct	**0.74**	**0.68**	0.81	**0.74**
Onset	PhenoPPIOrth	0.25	0.25	0.24	0.53
	Struct->Dis->HPO	0.07	0.06	0.10	0.49
	Binary SVMs	0.33	0.24	0.51	0.62
	Clus-HMC-Ens	0.35	0.27	0.48	0.58
	PHENOstruct	**0.39**	**0.31**	**0.52**	**0.64**

The performance is evaluated using macro AUC and protein-centric F-max, Precision and Recall (as defined above) on the complete HPO graph (i.e. true-path rule is applied to annotations and predictions).

**Table S4.** Comparison between PHENOstruct vs. other methods only leaf terms.

Subontology	Method	F-max	Precision	Recall	mac-AUC
Organ	PhenoPPIOrth	0.03	0.05	0.03	0.51
	Struct->Dis->HPO	0.01	0.01	0.02	0.50
	Binary SVMs	0.20	0.19	0.22	0.66
	Clus-HMC-Ens	0.08	0.04	0.31	0.64
	PHENOstruct	**0.30**	**0.21**	**0.50**	**0.77**
Inheritance	PhenoPPIOrth	0.11	0.15	0.09	0.55
	Struct->Dis->HPO	0.01	0.01	0.02	0.46
	Binary SVMs	0.69	0.62	0.78	0.71
	Clus-HMC-Ens	0.72	0.63	**0.84**	0.73
	PHENOstruct	**0.74**	**0.68**	0.82	**0.74**
Onset	PhenoPPIOrth	0.19	0.20	0.17	0.52
	Struct->Dis->HPO	0.03	0.02	0.14	0.49
	Binary SVMs	0.29	0.21	0.47	0.62
	Clus-HMC-Ens	0.28	0.21	0.43	0.58
	PHENOstruct	**0.35**	**0.28**	**0.48**	**0.68**

The performance is evaluated by using the exact annotations (i.e. only leaf terms) as ground truth. In other words, true-path rule is not applied. Performance is presented using macro AUC and protein-centric F-max, Precision and Recall as defined above.

**Table S5.** Validation of false positives in the Organ subontology.

Gene	HPO term	PubMed ID	Evidence
DKC1	Postnatal growth retardation	PMID: 10583221	“Hoyeraal-Hreidarsson (HH) syndrome is a multisystem disorder **affecting boys** characterized by aplastic anaemia (AA), immunodeficiency, microcephaly, cerebellarhypoplasia and **growth retardation**…We therefore analysed the **DKC1** gene in two HH families. In one family a nucleotide change at position 361(A–>G) in exon 5 was found in both affected brothers; in the other family a nucleotide change at position 146(C–>T) in exon 3 was found in the affected boys…”
PEX13	Neonatal hypotonia	PMID:12897163 (mouse)	“…In the studies reported here, we crossed these mice with transgenic mice that express Cre recombinase in all cells to generate progeny with ubiquitous disruption of **Pex13**. The mutant pups exhibited many of the clinical features of Zellweger syndrome patients, including intrauterine growth retardation, severe **hypotonia**, failure to feed, and neonatal death…”
PEX13	Retinal dystrophy	PMID:10441568, PMID: 10332040	“…The clinical course of patients with the NALD and IRD presentation is variable and may include developmental delay, hypotonia, liver dysfunction, sensorineural hearing loss, **retinal dystrophy** and vision impairment…” (UniProt entry for **PEX13**)
RPE65	Retinal dystrophy	PMID: 23878505	“…These results strongly suggest that causal mutations in **RPE65** are responsible for **retinal dystrophy** in the affected individuals of these consanguineous Pakistani families…”
BEST1	Retinitis pigmentosa	PMID: 19853238	“…Missense mutations in a retinal pigment epithelium protein, **bestrophin-1**, cause **retinitis pigmentosa**…”
PRPF6	Retinitis pigmentosa	PMID: 21549338	“…A missense mutation in **PRPF6** causes impairment of pre-mRNA splicing and autosomal-dominant **retinitis** **pigmentosa**…”
SNRNP200	Retinitis pigmentosa	PMID: 19878916	“…Autosomal-dominant **retinitis pigmentosa** caused by a mutation in **SNRNP200**, a gene required for unwinding of U4/U6 snRNAs…”
ORC1	Emphysema	PMID: 22333897	“…Four individuals were deceased: two siblings with mutations in **ORC1**, of which one passed away at the age of 3 months with a severe cortical dysplasia, pachygyria and ventricular enlargement, cranial suture stenosis, congenital **emphysema** of the lung, and absence of the pancreatic tail, in addition to the classical triad of MGS (microtia, patellar anomalies, and short stature)…”
CUL7	Hip dysplasia	PMID: 21396581	“A predisposing factor in **hip dysplasia** etiology is ligamentous laxity, presented in more than half of the patients with 3M syndrome described in the literature…3M syndrome is an autosomal recessive disorder. In 2005, using an homozygosity mapping strategy, we have mapped the disease locus gene on chromosome 6p21.1 and identified mutations in Cullin 7 ( **CUL7**, KIAA0076) gene…While **CUL7** appears to be the major gene responsible for 3M syndrome accounting for 70% of our cases,…”
ACTB	Postnatal microcephaly	PMID: 22366783	“…Riviere *et al.* (2012) reported on 10 children with Baraitser-Winter syndrome and mutations in the **ACTB** gene. Six of the 10 had short stature; 6 of 9 evaluated had **postnatal microcephaly**;…” (OMIM entry for **ACTB**)
ACTB	Progressive hearing impairment	PMID:16685646 (mouse)	“…However, aging mice with *β*- **actin** or *γ*-actin deficient hair cells develop different patterns of **progressive** **hearing loss** and distinct pathogenic changes in stereocilia morphology, despite colocalization of the actin isoforms…”
MSH2	Gastrointestinal carcinoma	PMID: 8252616	“… **hMSH2** maps to human chromosome 2p22-21 near a locus implicated in **hereditary nonpolyposis colon** **cancer (HNPCC)**…These data and reports indicating that S.cerevisiae msh2 mutations cause an instability of dinucleotide repeats like those associated with HNPCC suggest that **hMSH2** is the **HNPCC** gene…”
MSH2	Malignant gastrointestinal tract tumors	PMID: 8252616	“… **hMSH2** maps to human chromosome 2p22-21 near a locus implicated in **hereditary nonpolyposis colon** **cancer (HNPCC)**…These data and reports indicating that S.cerevisiae msh2 mutations cause an instability of dinucleotide repeats like those associated with HNPCC suggest that **hMSH2** is the **HNPCC** gene…”
MSH2	Hereditary nonpolyposis colorectal carcinoma	PMID: 8252616	“… **hMSH2** maps to human chromosome 2p22-21 near a locus implicated in **hereditary nonpolyposis colon** **cancer (HNPCC)**…These data and reports indicating that S.cerevisiae msh2 mutations cause an instability of dinucleotide repeats like those associated with HNPCC suggest that **hMSH2** is the **HNPCC** gene…”

The columns “HPO term”, “PubMed ID” and “Evidence” provides the false positive prediction made by PHENOStruct for the given gene, PubMed ID of the literature that contains evidence which actually suggests that the prediction should be considered true and the excerpt from that literature which contains the evidence, respectively. We used the 25 false positive predictions for the 17 proteins that had the highest individual protein-centric precision and found evidence for 14 predictions. Two of the evidence comes from studies involving mice (indicated within parentheses with the PubMed ID)

**Table S6.** Performance of Binary SVMs in the Inheritance subontology.

Name	Freq.	Depth	AUC
Multifactorial inheritance	15	1	0.62
Polygenic inheritance	15	2	0.62
Mitochondrial inheritance	41	1	0.96
Sporadic	52	1	0.66
Somatic mutation	61	1	0.71
X-linked dominant inheritance	62	3	0.79
X-linked recessive inheritance	111	3	0.70
Heterogeneous	148	1	0.65
X-linked inheritance	198	2	0.78
Gonosomal inheritance	198	1	0.78
Autosomal dominant inheritance	1096	1	0.69
Autosomal recessive inheritance	1665	1	0.68

The macro AUC for the Inheritance subontology is 0.72. Terms are displayed in ascending order of frequency.

## Supplementary material

### Analysis of variant features

In this section we analyze the performance of the features generated from genetic variant data in detail. The macro AUC for the variants data is only 0.56 in the Organ subontology. However, 37 terms have an AUC equal to or above 0.9. As listed in the Table S1, 21 out of those 37 terms well-predicted by the variant data are terms related to cancer. But interestingly, only 53 out of 1796 of all the Organ subontology terms are related to cancer. This shows strong evidence that the genetic variant data are highly effective for predicting cancer related phenotype terms. Terms predicted with high accuracy by the literature features do not show a similar tendency (data not shown).

In the Inheritance subontology it was noticeable that the variant data are more effective for the categories with fewer annotations compared to the literature features (data not shown). The average number of annotations of the Inheritance subontology HPO categories with relatively higher AUC by variant features (compared to literature features) is only 46. This trend is also visible, albeit to a lesser extent, in the Organ and Onset subontologies as well; the corresponding numbers for the Organ and the Onset subontologies are 26 and 91, respectively. Furthermore, only mitochondrial inheritance term achieves an AUC above 0.9 with all data sources. However, with variant data alone, both mitochondrial inheritance and somatic mutation terms achieve AUCs above 0.9.

### Analysis of literature features

In order to identify the most important literature features, we looked at the weight vectors of the structured SVM model underlying PHENOstruct that was trained only on the literature features. Typically, the input space features with higher weight in the weight vector correspond to the features that are considered most important by the model for the given predictive task.

In the dual formulation of the Structured SVM, *α _ij_* values are defined for each pair of example *i* and structured output *j*. In order to calculate the weight vector for a specific HPO term *j* ( *W _j_*), we first identified the subset of input examples (i.e. proteins) that are annotated with the given term (referred to as *S _j_*). Then *W _j_* is the summation of *α _ij_* × *x _i_* where *x _i_* is the feature vector of example *i* and *x _i_* ∈ *S _j_*. Features with higher weights in the weight vector *W _j_* correspond to the features that were considered most informative by the model for the task of predicting the term *j*.

We trained PHENOstruct on literature features and computed the weight vectors as described above. Then we ranked the literature features by their weights and examined the top-100 literature features. In the Organ subontology we analysed the top-100 literature features with respect to the 8 HPO terms that have individual AUCs above 0.9. For those 8 terms the union set of top-100 features is composed of 107 unique tokens. By far, the majority (>70%) of these tokens are genes/ proteins/ protein complexes/ pathways names. Another 12 tokens are disease/phenotype names ( Table S2).

### Additional methods

We describe here the results of experiments that we conducted with two additional methods.

*SSVM* → *disease* → *HPO method* This is an indirect method that first predicts gene-disease associations and then maps them to HPO terms using associations available on the HPO website. This method uses the same input space data as PHENOstruct and learns a structured SVM using the same methodology. Using this model it predicts diseases along with confidence scores for unseen genes. Subsequently, the predicted scores for disease terms are directly transferred to all the HPO terms associated with those diseases ( Figure S1). When multiple diseases are associated with a single HPO term, scores are accumulated. It is surprising that this method shows mediocre performance ( Table S3). One of the main reasons for this is the low performance of the underlying SSVM for predicting disease terms (average AUC of 0.64), which consequently affects the accuracy of predicted HPO terms.

*PhenoPPIOrth* We also evaluated the performance of PhenoPPIOrth (Wang *et al.*, 2013). PhenoPPIOrth is a computational tool that can predict a set of diseases for a given human gene. Specifically, it predicts OMIM disease terms for human genes using protein-protein interaction and orthology data. Then it also maps the predicted OMIM terms to HPO terms using the disease-HPO mapping available in the HPO website ^11^. We downloaded the pre-computed preditions from the PhenoPPIOrth website. Compared to PHENOStruct, PhenoPPIOrths performance was quite low (see Table S3). It is important to note that PhenoPPIOrth makes predictions for only a subset of proteins with respect to all three ontologies (1487, 175 and 155 in Organ, Inheritance and Onset subontologies, respectively). One of the main reasons is that HPO annotations are generated using three sources: OMIM, Orphanet and DECHIPER but PhenoPPIOrth predicts only OMIM terms.

### Performance measures

We use *term-centric* AUC or macro AUC as our primary evaluation measure for reporting results. In addition, we use several *protein-centric* measures. Protein-centric precision and recall at a given threshold *t* are defined as

$$Pr_{pc}(t)\;=\;\frac{1}{N}\sum_{i=0}^{N}\frac{TP{\left(t\right)}_{i}}{TP{\left(t\right)}_{i}\;+\;FP{\left(t\right)}_{i}},$$

$$Rc_{pc}(t)\;=\;\frac{1}{N}\sum_{i=0}^{N}\frac{TP{\left(t\right)}_{i}}{TP{\left(t\right)}_{i}\;+\;FN{\left(t\right)}_{i}},$$

where *TP*( *t*) _*i*_, *FP*( *t*) _*i*_ and *FN*( *t*) _*i*_ are the number of true positives, number of false positives and number of false negatives w.r.t. protein *i* at threshold t. Now we can define protein-centric F-max as

$$F_{pc}-max\;=\;\underset{t}{\text{max}}\frac{2Pr_{pc}(t)Rc_{pc}(t)}{Pr_{pc}(t)\;+\;Rc_{pc}(t)}.$$

### Complete results

In this section we present performance of all five methods using several performance measures.

### Validating false positives

First we ranked the test proteins in descending order of the protein-centric precision of their Organ subontology predictions made by PHENOstruct. Then we retrieved the 25 false positive predictions for the top 17 proteins in that list. Next, we performed online searches using the pair of protein name and phenotype name as the query for the search engine. This resulted in a list of publications for each false positive prediction. Then we manually extracted the excepts from those papers that contained supporting evidence that suggests the particular false positive is in fact correct. Using this manual process we found evidence for 14 of the 25 false predictions considered for this study (see Table S5). For two of the cases the evidence comes from studies involving mice (indicated within parentheses with the PubMed ID). Overall success of this study strongly suggests that the performance of PHENOstruct is under-estimated due to the incompleteness of the current gold standard.
