# Supplementary material for: PHENOstruct: Prediction of human phenotype ontology terms using heterogeneous data sources
Source: F1000Res. 2015 Jul 16;4:259. [Version 1] doi: 10.12688/f1000research.6670.1 (PMC4722686; doi:10.12688/f1000research.6670.1)
Supplement: Peer review history

# Peer review: Referee response for version 1

**DOI:** 10.5256/f1000research.7166.r9995

**Published:** 2015-08-28

**Authors:** Shaillay Dogra

**Affiliations:** 1 Vishuo BioMedical Pte Ltd, Singapore, Singapore

A well written article with detailed methodology towards mapping genes to diseases. The method proposes to overcome the limitations of traditional approaches, which take single-label at a time. Author's approach uses structured prediction that takes into account related set of labels.

Some general points for authors consideration:

1. What is a possible, direct application of this method? How can this be integrated with other software tools or called as a service, for example?
2. Different data sources have been used of which protein-protein interactions, for example, is a noisy one with false-interactions reported via experimental methods like co-precipitation and yeast-two-hybrid systems. Can the author's comment on how the quality of data affects their approach? How to deal with noisy data sources or reduce the weight of contribution from that particular data source?
3. How easy is to update this, for example, integrating latest PubMed abstracts? What's the pipeline or process to do so?

Some specific points for authors to consider:

1. Why do we expect more genes to be disease-causing? Is it just a general line of reasoning that functional genes should cause an aberrant phenotype if they do not work properly?
2. Perhaps the authors can expand a bit more on the problem formulation - "therefore, it is important to explore the feasibility..."
3. Under "Approach", perhaps the authors can explain a bit on HMC for the benefit of the readers. Also, a bit more on "structured prediction/learning" in layman terms or illustrated with an example can help the reader grasp the concept.
4. Authors can consider simplifying this into shorter sentences for easy grasp - "An alternate approach is to use a single classifier..."
5. Under "HPO annotations" - for general understanding, could the authors tell more about why they 'removed terms that were not annotated to 10 or more genes.'
6. Under "Literature" - abstracts extracted are from 2013 and not up to date with 2015.
7. Under "Literature" - for general understanding, could the authors tell more about why they 'filtered to keep only the low frequency words'.
8. Under "variants" - does this data from Uniprot covers data sources like clinvar, dbGaP, GWAS studies etc?
9. Under "Models", for the general understanding of the reader could the authors expand on what is meant by a structured model?
10. Figure 3 - last part of panel is barely legible.
11. Under "Evaluation" could the authors expand on what's implied by term-centric and protein-centric, F-max?
12. "The human mitochondrial DNA... Due to this, and the relative..." - I am not sure if this is the reason.
13. Authors can consider simplifying this into shorter sentences for easy grasp - "As a potential improvement to PHENOstruct..."
14. I really liked the way authors dealt with "validating false positives" (text and table S5).
15. Figure S1 - last part of panel is almost illegible.
16. Supplementary material, "Performance Measures" - for the benefit of the readers, what does F-max mean in a literal, intuitive sense?
17. Table S4 -- for the benefit of the readers, what is 'true-path rule', 'ground truth', 'macro AUC'...?

I have read this submission. I believe that I have an appropriate level of expertise to confirm that it is of an acceptable scientific standard.

# Peer review: Referee response for version 1

**DOI:** 10.5256/f1000research.7166.r9567

**Published:** 2015-08-14

**Authors:** Peter Robinson

**Affiliations:** 1 Institute for Medical and Human Genetics, Humboldt-Universität, Berlin, Germany

The authors present a clever strategy for using a machine learning approach to predict associations between genes and human phenotype ontology (HPO) terms. The HPO, as many other ontologies like the Gene Ontology, has a hierarchical structure such that annotations to HPO terms are inherited up the structure of the ontology. For instance, if we say that a patient has "ventricular septal defect", we implicitly annotate the patient to all of the ancestor terms of "ventricular septal defect" such as "abnormality of the ventricular septum". This creates a problem for naive machine learning approaches that make HPO term/Gene association predictions one at a time. If a prediction is YES for "ventricular septal defect" but NO for "abnormality of the ventricular septum", then the result is mutually inconsistent. A number of machine learning algorithms have emerged to tackle this problem, including one that the authors previously learned for an analogous project with Gene Ontology predictions. In general, the paper is very well done and it is likely to be accessible to a wide audience because it is well written. The topic of HPO annotation prediction is very new, and there is no other published work on the topic that I am aware of at present, although a number of groups, including the authors, participated in a CAFA competition at the 2014 ISMB.

Suggestions:

1. The authors should cite GeneMania ( **The GeneMANIA prediction server: biological network integration for gene prioritization and predicting gene function** Warde-Farley et al, 2010, NAR).
2. The authors should state the version of the HPO and the HPO annotation data they used. In the meantime, the number of annotations has increased substantially, and a number of improvements to the HPO structure have been made (for instance, the Organ abnormality term has been renamed to Phenotypic abnormality).
3. It would be nice to have a little more self-contained explanation about some of the methods employed, such as Clu-HMC-Ens.
4. The authors observed an excellent AUROC score for mitochondrial inheritance, and speculate that the reason is that the mitochondrial genome is well studied. I suspect that the true reason might be that mitochondrial genes have a very specialized functional profile (energy etc) that is much more homogeneous than say "autosomal recessive".
5. The Mammalian phenotype ontology is not only used to annotate the rat genome, but is the major tool used to annotate the mouse genome, and is an extremely useful resource used now by the International Mouse Phenotyping Consortium the Mouse Genome Informatics group, and many others. This should be added to the text.

I have read this submission. I believe that I have an appropriate level of expertise to confirm that it is of an acceptable scientific standard.
